# Oral PrEP Consultations Among Adolescent Girls and Young Women in Kisumu County, Kenya: Insights from the DREAMS Program

**DOI:** 10.1007/s10461-022-03590-z

**Published:** 2022-01-31

**Authors:** Craig J. Heck, Sanyukta Mathur, Habel Alwang’a, Oluoch-Madiang’ Daniel, Rael Obanda, Mophine Owiti, Jerry Okal

**Affiliations:** 1 Department of Epidemiology, Mailman School of Public Health, Columbia University, 722 West 168th Street, New York, NY 10032, USA; 2 Population Council, Washington, DC, USA; 3 PATH, Kisumu, Kenya; 4 Population Council, Nairobi, Kenya

**Keywords:** AGYW, PrEP, DREAMS, HIV prevention, Implementation science, Kenya

## Abstract

Although Kenya nationally scaled up oral pre-exposure prophylaxis (PrEP) in May 2017, adolescent girls’ (AG, aged 15–19 years) and young women’s (YW, aged 20–24 years) PrEP use remains suboptimal. Thus, we analyzed PrEP consultations—interactions with a healthcare provider about PrEP—among Kenyan AGYW. In April-June 2018, AGYW enrolled in DREAMS in Kisumu County, Kenya self-reported their HIV-related knowledge, behaviors, and service use. Among HIV negative, sexually active AG (n = 154) and YW (n = 289), we examined associations between PrEP eligibility and PrEP consultations using prevalence ratios (PR, adjusted: aPR). Most AG (90.26%) and YW (94.12%) were PrEP-eligible due to inconsistent/no condom use, violence survivorship, or recent sexually transmitted infection symptoms. Between PrEP-eligible AG and YW, more YW were ever-orphaned (58.09%), ever-married (54.41%), ever-pregnant (80.88%), and out of school (78.31%); more PrEP-eligible YW reported PrEP consultations (41.18% vs. 24.46%, aPR = 1.51 [1.01–2.27]). AG who used PEP (post-exposure prophylaxis) reported more consultations (aPR = 5.63 [3.53–8.97]). Among YW, transactional sex engagers reported more consultations (58.62% vs. 39.09%, PR = 1.50 [1.06–2.12]), but only PEP use (aPR = 2.81 [2.30–3.43]) and multiple partnerships (aPR = 1.39 [1.06–1.82]) were independently associated with consultations. Consultations were lowest among those with 1 eligibility criterion (AG = 11.11%/YW = 27.18%). Comparatively, consultations were higher among AG and YW with 2 (aPR = 3.71 [1.64–8.39], PR = 1.60 [1.07–2.38], respectively) or ≥ 3 (aPR = 2.51 [1.09–5.78], PR = 2.05 [1.42–2.97], respectively) eligibility criteria. Though most AGYW were PrEP-eligible, PrEP consultations were rare and differed by age and vulnerability. In high-incidence settings, PrEP consultations should be conducted with all AGYW. PrEP provision guidelines must be re-assessed to accelerate AGYW’s PrEP access.

## Introduction

Despite geopolitical commitments, increased donor investments, and novel program delivery, adolescent girls (AG, aged 15–19 years) and young women (YW, aged 20–24 years) remain at disproportionate risk of HIV acquisition [1]. Globally, AGYW make up 75% of annual seroconversions among 15- to 24-year-olds, with an estimated 7000 AGYW seroconverting weekly. Despite being only 10% of sub-Saharan Africa’s population, AGYW account for 20% of the region’s seroconversions [2]. In Kenya, AGYW’s prevalence (2.6%) and incidence (approximately 12,500 seroconversions annually) are approximately double that of their male counterparts [3]. This gendered disparity is driven by life transitions, such as orphanhood [4–6], early marriage and pregnancy [6–11], and school dropout [12, 13]; not knowing a partner’s HIV status [14, 15]; behaviors, like substance use [7, 16–18], transactional sex [7, 19], multiple partnerships [7, 18], and inconsistent/no condom use [20, 21]; and experiences, including sexually transmitted infections (STIs) [12, 21] and survivorship of physical and/or sexual violence from intimate [22–25] and/or non-partners [22, 24, 25].

Oral pre-exposure prophylaxis (PrEP) has become a primary focus of HIV prevention efforts because it is safe and highly efficacious [26]. Given their risk, AGYW are a priority population for PrEP, yet research suggests that PrEP access, uptake, adherence, and continuation is low among AGYW [15, 27–30]. The HIV prevention field experienced a paradigm shift in May 2017, when—after a national pilot program [31]—PrEP became widely available to Kenyans, including AGYW. As of October 2021, an estimated 127,500 Kenyans have cumulatively initiated oral PrEP [32]. However, due to aggregated data sources, it is difficult to ascertain how many of these initiators are AGYW.

Along with demand, PrEP’s utility is conditional on its availability and provision. Per Dunbar et al., the oral PrEP cascade underscores that there must be synergy between service availability and screening, offer, and initiation processes for the total target population (i.e., “all seronegative [AGYW] at risk for HIV who could benefit from PrEP”) to start PrEP [33]. Because PrEP initiation, adherence, and continuation among AGYW is low [15, 27–30] and affected by low perceived risk and concerns of pill burden, partner disapproval, side effects, and stigma [34–38], researchers, program planners, and implementers have concentrated on the cascade’s downstream factors. While warranted, emphasis on these downstream elements has led to a dearth of information on upstream processes, knowledge that would help illuminate if and how vulnerable AGYW are being identified and offered PrEP services.

In this paper, we aimed to investigate AGYW’s PrEP eligibility and interactions with PrEP providers, insights that can inform service access, linkages, retention, and provision. First, we used the Ministry of Health’s (MOH) PrEP eligibility criteria to identify the total target population among AGYW enrolled in DREAMS (Determined, Resilient, Empowered, AIDS-Free, Mentored, Safe) in Kisumu County, Kenya. Then, among this total target population, we examined associations between individual and cumulative MOH PrEP eligibility criteria and PrEP consultations to identify correlates and trends. These findings have the potential to inform strategies that increase AGYW’s access to PrEP services; improve providers’ ability to identify vulnerable clients; strengthen service delivery; and, subsequently, reduce AGYW’s HIV acquisition risk.

## Methods

### Study Population and Sample

For this analysis, we used data on DREAMS enrollees who participated in the DREAMS Implementation Science study. Additional details regarding DREAMS and its methods are provided elsewhere [1, 39]. Briefly, DREAMS uses community-based safe space platforms to engage AGYW in activities related to positive sexual behaviors, self-efficacy, socioeconomic approaches, and sexual and reproductive health. In Kenya, DREAMS operates in seven counties with high HIV incidence—namely Kisumu, Homa Bay, Migori, Siaya, Nairobi, Kiambu, and Mombasa [40]. The DREAMS Implementation Science study was conducted in Kisumu County, an urban/peri-urban setting neighboring Lake Victoria, where the HIV prevalence among adults (aged 15–64 years) is 17.5%, almost four-times the national average of 4.9% [41].

Based on program eligibility criteria, DREAMS staff enrolled 15- to 24-year-old females into the program if they lived and intended to stay in the program catchment area. Using structured survey instruments, study personnel collected enrollees’ self-reported knowledge, attitudes, practices, and experiences related to HIV; sexual and reproductive health; and healthcare utilization at two survey rounds [42, 43]. Longitudinal findings on this cohort of AGYW are presented elsewhere [44]. Since PrEP was not available at round 1 (October–November 2016), we used round 2 (April-June 2018) data for this analysis. Because PrEP use is conditional on HIV seronegativity and sexual activity, we excluded AGYW from this analysis if they were living with HIV or sexually inexperienced (i.e., reported never having sex) or inactive (i.e., reported ever having sex but no reported sex partner in the last 12 months).

### Measures

The variable definitions for all study measures are presented in Table 1.

#### PrEP Eligibility

Using Kenya’s MOH PrEP eligibility criteria [45, 46], we identified participants “at substantial risk of acquiring HIV.” These criteria included attempting to conceive while in a serodiscordant relationship, recurrent drug or alcohol use during sex, transactional sex engagement, recurrent post-exposure prophylaxis (PEP) use, sex with a partner of unknown HIV status, multiple sexual partnerships, recent STI experience, survivorship of intimate partner violence (IPV) or gender-based violence (GBV), and inconsistent or no condom use. Per MOH guidelines, these eligibility criteria are framed within a 6-month recall period. Due to the wording used in the DREAMS survey, most eligibility criteria were measured as occurring in the last 12 months (Table 1). Like the MOH guidelines, if a participant had at least one criterion, we considered them eligible for PrEP and, thus, a member of the total target population.

#### Sample Characteristics

For stratification purposes, we dichotomized age to differentiate between AG (aged 15–19 years) and YW (aged 20–24 years) and examined characteristics that could affect participants’ risk, such as socioeconomic position; orphanhood, marital, pregnancy, and schooling status; perceived HIV risk; travel outside the community; and location. When investigating the outcome, we also explored clinic/hospital travel time, which we used to assess differential service access (Table 1).

#### Outcome

We analyzed participants’ reports of PrEP consultations using a composite measure that captured interactions with a PrEP provider that occurred ever or at the last clinic visit (Table 1). We included “ever” responses because the survey was administered (April-June 2018) approximately one year after PrEP scale up (May 2017). In the DREAMS program, 18–24-year-olds were targeted for PrEP provision, meaning those aged 18–19 years in the AG age group had a greater opportunity for PrEP consultations than those aged 15–17 years. To account for this difference, we created a covariate (program targeting) to signify AG who were aged < 18 years after adding 1 year to their baseline age (67.53% among AG). We took this approach to account for the time difference between Round 1 data collection and national scale-up of PrEP, approximately 6–7 months. Though PrEP consultations did not differ across program targeting categories among PrEP-eligible AG (24.44% vs. 24.47%, Chi-square = 0.00, p = 0.998), we included it as a covariate in all analyses that examined the outcome among AG to control for any residual effects.

### Statistical Analysis

For all analyses, we used an age-stratified approach and α = 0.05 to assess statistical significance. When generating cross-tabulations, we calculated p-values using Pearson’s Chi-squared Test and Fisher’s Exact Test, when appropriate (i.e., an expected cell count is < 5). We also examined differences in medians among YW using the Wilcoxon Rank-Sum Test; for AG, we used the van Elteren Test [47], a stratified extension of the Wilcoxon Rank-Sum Test, to account for differential program targeting.

For regression analyses, we used generalized linear models (log link, Poisson family, Huber-White sandwich variance estimator) to generate unadjusted and adjusted prevalence ratios (PR and aPR, respectively). We used this approach because when the outcome is common (i.e., > 10% prevalence), prevalence ratios better approximate relative risk than odds ratios [48–50]. Using Spearman’s correlation (cut-off of 0.80 [51]) and variance inflation factors (cut-off of 10 [52]), we concluded that multicollinearity did not affect any of the multivariable models (Supplemental Table 1).

When investigating PrEP consultation correlates, we fitted a preliminary adjusted model using all characteristics and PrEP eligibility criteria with an unadjusted p < 0.05. Then, we used backward selection to remove the PrEP criterion with the largest p-value until all PrEP eligibility criteria were statistically significant; characteristics remained in the adjusted model regardless of their adjusted p-value.

When analyzing the relationship between PrEP consultations and cumulative of PrEP eligibility criteria, we trichotomized the criteria totals using the median as the midpoint. We conducted all analyses using Stata v16.

### Ethics

All respondents provided written informed consent or assent and parental consent; emancipated minors (i.e., married, pregnant, or a parent) provided consent. Participants were compensated 300 Kenyan Shillings (approximately USD $3) for their time. Ethical approval of study protocols and procedures was obtained from the institutional review boards at the Population Council and Kenyatta National Hospital/University of Nairobi Ethics and Research Committee and National Commission for Science, Technology, & Innovation (NACOSTI). Columbia University provided a “not human subjects research” designation for this secondary data analysis (#AAAS8976).

## Results

Of the 736 DREAMS enrollees interviewed at round 2, we excluded 39.81% (293/736) because they were living with HIV or unsure/non-responsive about their HIV status (n = 37), sexually inexperienced (n = 232), or sexually inactive (n = 24), resulting in an initial sample of 443: 154 AG and 289 YW.

### Identification of the Total Target Population

Table 2 highlights the frequency of each PrEP criterion. Overall, AG and YW had similar proportions for most PrEP eligibility criteria. However, compared with YW, AG had fewer reports of alcohol use during sex (2.60% vs. 10.03%, Chi-Square = 8.06, p = 0.005) and inconsistent/no condom use (74.03% vs. 89.27%, Chi-Square = 17.36, p < 0.001). In total, 90.26% of AG (139/154) and 94.12% of YW (272/289) reported at least one criterion, making them eligible for PrEP.

### Characteristics Associated with PrEP Eligibility Among AG and YW

Table 3 details characteristic differences by age group and PrEP eligibility. More PrEP-eligible AG reported high perceived HIV risk than PrEP-ineligible AG (24.46% vs. 0.00%, Fisher’s p = 0.043). All 5 AG who were currently pregnant were PrEP-eligible (data not shown).

Compared with their ineligible counterparts, more PrEP-eligible YW were ever-married (54.41% vs. 5.88%, Chi-Square = 15.09, p < 0.001), ever-pregnant (80.88% vs. 29.41%, Fisher’s p < 0.001), and out of school (78.31% vs. 41.18%, Fisher’s p = 0.002). Of the 18 currently pregnant YW, all were PrEP-eligible (data not shown). When a multivariable model (Model I) was fit with marital, pregnancy, and schooling status, an ever-pregnant status (aPR = 1.15 [1.02–1.30]) was the only factor significantly greater among PrEP-eligible YW.

Between PrEP-eligible AG and YW, fewer AG were ever-orphaned (41.73% vs. 58.09%, Chi-Square = 9.88, p = 0.002), ever-married (16.55% vs. 54.41%, Chi-Square = 54.29, p < 0.001), ever-pregnant (38.85% vs. 80.88%, Chi-Square = 73.14, p < 0.001), and out of school (47.48% vs. 78.31%, Chi-Square = 40.10, p < 0.001). When examined together in a multivariable model (Model II), ever-married (aPR = 0.48 [0.30–0.77]), ever-pregnant (aPR = 0.52 [0.38–0.72]), and out-of-school status (aPR = 0.72 [0.56–0.94]) remained significant.

### Examination of PrEP Consultation Correlates Among PrEP-eligible AG and YW

Table 4 presents findings related to PrEP consultations. Of 139 PrEP-eligible AG, only 24.46% (34/139) reported PrEP consultations, while 41.18% of PrEP-eligible YW (112/272) reported consultations. Compared with AG, YW reported more PrEP consultations (aPR = 1.68 [1.15–2.47], controlling for program targeting), even after controlling (Model III) for intergroup differences (orphanhood, marital, pregnancy, and schooling status and program targeting: aPR = 1.51 [1.01–2.27]).

Among PrEP-eligible AG, travel outside the community was the only characteristic associated with PrEP consultations. Specifically, PrEP-eligible AG who frequently (≥ 1 per month) traveled outside the community reported more PrEP consultations (aPR = 2.04 [1.13–3.68], controlling for program targeting) than infrequent (≤ 1 × year) travelers. Regarding PrEP eligibility criteria, PrEP-eligible AG who were recurrent PEP users reported more PrEP consultations (93.75% vs. 15.45%, *z* = 8.15, p < 0.001). After controlling for travel outside the community and program targeting, this association remained (aPR = 5.63 [3.53–8.97], [Model IV]).

For PrEP-eligible YW, PrEP consultations were not associated with socioeconomic position; orphanhood, marital, pregnancy, and schooling status; perceived HIV risk; travel outside the community; location; or clinic/hospital travel time. PrEP consultations were higher among YW who reported transactional sex (58.62% vs. 39.09%, Chi-Square = 4.08, p = 0.043), recurrent PEP use (97.22% vs. 32.63%, Chi-Square = 53.81, p < 0.001), and multiple sexual partners (62.79% vs. 37.12%, Chi-Square = 9.85, p = 0.002). The initial adjusted model included these three criteria (Model V): after the removal of transactional sex per our model building approach, recurrent PEP use (aPR = 2.81 [2.30–3.43]) and multiple sexual partnerships (aPR = 1.39 [1.06–1.82]) were independently associated with PrEP consultations (Model VI).

### PrEP Consultations Among PrEP-Eligible AG and YW by Cumulative PrEP Eligibility Criteria

**Fig. 1:**
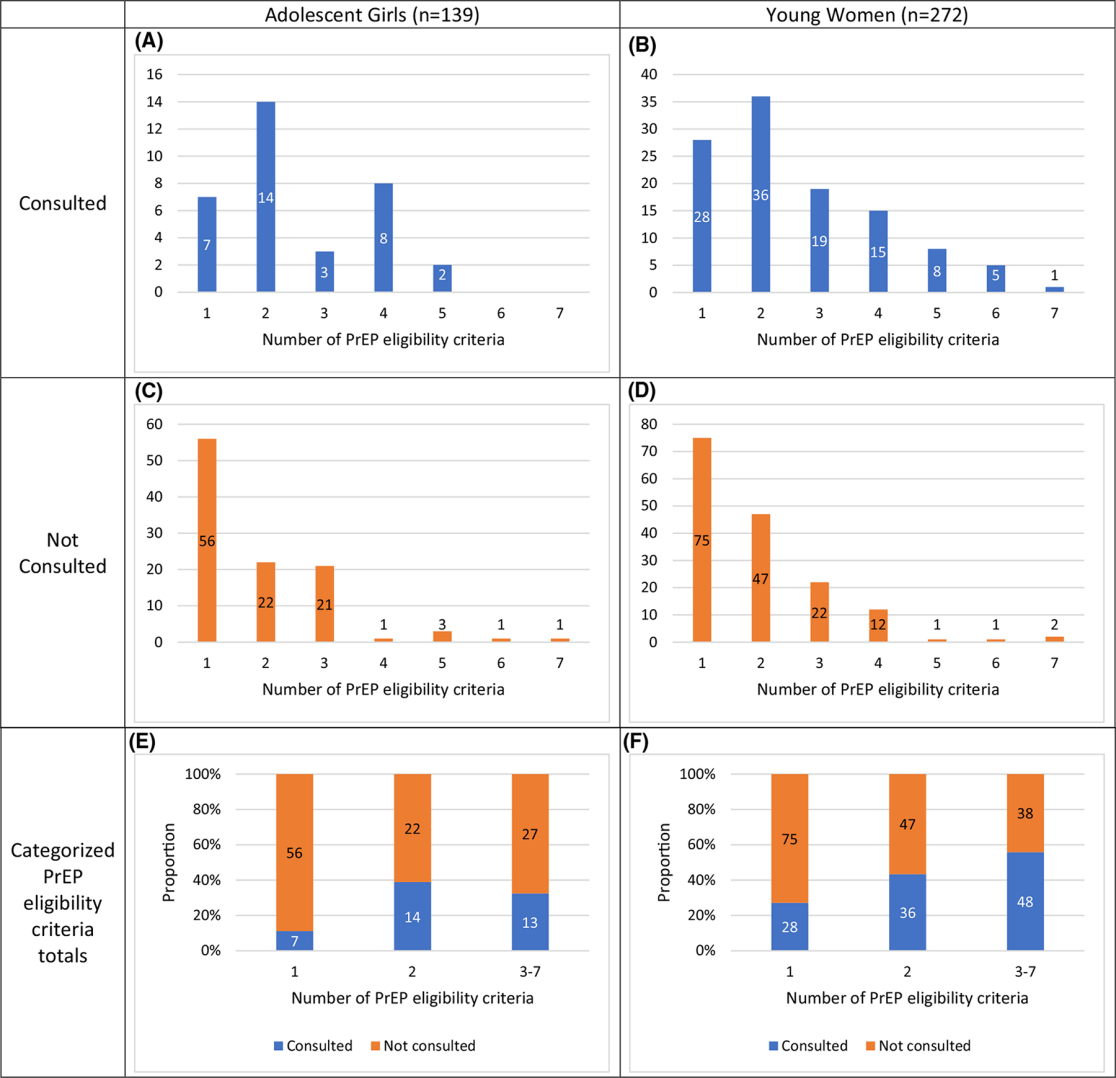
Relationship between cumulative PrEP eligibility criteria and PrEP consultations among PrEP-eligible AGYW. *PrEP* pre-exposure prophylaxis, *AGYW* adolescent girls and young women. Numbers within each bar represent the number of respondents

Figure 1 showcases the relationship between PrEP consultations and the cumulative number of PrEP eligibility criteria. The median number of criteria among both PrEP-eligible AG and YW was 2 (IQR: 1–3). While the median number of criteria by PrEP consultation category was similar between AG (Consulted: 2 [IQR: 2–4], Not Consulted: 1 [IQR: 1–3]) and YW (Consulted: 2 [IQR: 1.5–4], Not Consulted: 2 [IQR: 1–2]), there were within-group differences for both AG (*z* = − 3.204, p = 0.001) and YW (*z* = − 4.281, p < 0.001). In absolute terms, the number of consultations decrease in AG and YW after 2 criteria (Fig. 1A, B). Relatively, PrEP consultations were low (i.e., < 50%) for most PrEP eligibility criteria totals in each age group (Fig. 1A–D). However, though there was no apparent relationship for AG (Fig. 1A, C), the proportion of PrEP consultations among YW gradually increased as the number of PrEP eligibility criteria increased (Fig. 1B, D).

Using the categorized totals, AG with 2 or ≥ 3 PrEP eligibility criteria were more likely to report a PrEP consultation (aPR = 3.71 [1.64–8.39] and aPR = 2.51 [1.09–5.78], respectively) than AG with 1 criterion (Fig. 1E)—after controlling for differential program targeting and travel outside the community, which differed significantly by outcome status (Table 2). Similarly, YW with 2 (PR = 1.60 [1.07–2.38]) or ≥ 3 (PR = 2.05 [1.42–2.97]) criteria were more likely to report PrEP consultations (Fig. 1F).

## Discussion

To our knowledge, this analysis is one of the first to use an age-stratified approach to examine AGYW’s connection to PrEP services by assessing the Kenya MOH’s PrEP eligibility criteria. Almost all AGYW had ≥ 1 MOH PrEP eligibility criteria and were, therefore, eligible for PrEP. However, few PrEP-eligible AGYW reported PrEP consultations, and consultations were significantly lower among AG than YW, even after accounting for intergroup sociodemographic differences (orphanhood, marital, pregnancy, and schooling status) and AG’s differential program targeting. For correlates of PrEP consultations, AG who were PEP users reported more screenings, and YW who used PEP and had multiple sex partners reported greater screenings. Though low overall, reports of PrEP consultations were higher among AG and YW with 2 or 3–7 PrEP eligibility criteria compared with 1 criterion. These findings imply that despite their high HIV risk, AGYW’s access to PrEP was limited, necessitating operational changes and capacity strengthening efforts to improve providers’ ability to identify clients with high HIV vulnerability and increase AGYW’s access to PrEP services.

By reporting one or more MOH criteria, almost all AGYW participants were considered at risk of HIV acquisition, with the three most common criteria being inconsistent/no condom use, ongoing IPV/GBV, and STI symptoms. Along with a link to HIV risk [20, 22, 23, 25, 53, 54], these risk factors are fueled by gender and power inequality [55–58]. In gender-inequitable environments, AGYW are more likely to encounter failed condom negotiations and violence perpetuated by intimate partners and/or strangers—which expose AGYW to STIs and mediate other negative health and psychological outcomes [17, 22, 58, 59]. Thus, activities to increase AGYW’s PrEP access must be complemented with efforts to engage males and couples in dialogue about PrEP, gender equality, and egalitarian relationships. Evidence from South Africa, Tanzania, and Zimbabwe support that as relationship dynamics equalize and male partners’ knowledge of PrEP increase, AGYW’s PrEP-related outcomes (e.g., initiation, adherence, and continuation) improve, too [35, 60, 61].

Among PrEP-eligible AG and YW, numerous characteristics were associated with HIV vulnerability. Albeit only in the unadjusted analyses, we discovered that more PrEP-eligible than -ineligible AG had high perceived HIV risk, a promising finding since risk perception influences care-seeking behaviors [62]. Consistent with the current evidence, we also learned that more PrEP-eligible YW were ever-married and out of school than PrEP-ineligible YW [13, 53], reinforcing their continued focus in AGYW HIV prevention programming. Relatedly, pregnancy history was independently associated with substantial HIV risk [17, 37, 63] in YW, underscoring the need to integrate PrEP services into sexual and reproductive services (e.g., sexual health, family planning, maternal and child health) to avoid missed opportunities and ensure comprehensive coverage [15, 17, 64, 65]. When compared with PrEP-eligible AG, many of these factors (out-of-school, ever-married, ever-pregnant status) plus orphanhood were higher in PrEP-eligible YW. Considering the age difference, YW have greater opportunity to experience these life transitions, so these findings are not unexpected. Novel analytic methods, such as latent class analysis, should be used in the future to illuminate how synergies between AGYW’s multiple vulnerabilities manifest and relate to HIV risk and service access and use.

Despite ubiquitous risk, most AGYW did not report PrEP consultations. Evidence from other settings and contexts offer some potential logistical explanations for this disparity: limited human resources, suboptimal processes, and high client volumes [66–68]. Qualitative research from Kenya shows that heavy workloads truncate client-provider interactions and impact service provision, causing providers to focus solely on the client’s direct request rather than offer additional services or inquire about other health concerns [29]. Additionally, evidence from South Africa and the United States support that providers’ PrEP knowledge, awareness, and assumptions [69, 70] can affect service provision. Findings from Tanzania reinforce that provider- and facility-level capacity strengthening activities are also vital to ensuring providers are ready, willing, and able to offer PrEP services [71]. To confirm PrEP services are well-integrated and not a burden on health care providers or clients, closer examination of facility-level staffing, workflows, and procedures is recommended.

Despite similar risk distributions, PrEP consultations—though low overall—were significantly lower in AG than YW, even after controlling for differences in program targeting among AG. Provider attitudes could have contributed to this difference [72, 73]. Research from Kenya suggests that providers are concerned about prescribing AGYW oral PrEP because it could lead to risky behaviors, such as multiple sex partners and condomless sex [74, 75], a sentiment corroborated by qualitative interviews with providers in Tanzania [71]. Kenyan providers have also expressed that AG should be offered abstinence counseling rather than PrEP, AG lack the responsibility required for PrEP adherence, and providing PrEP to AG would cause community backlash [74, 75], a key reason why DREAMS chose not to target 15–17-year-olds for PrEP provision. Alternatively, consultations might have been lower because AG were uncomfortable disclosing behaviors and experiences due to provider mistrust, fear of sexuality-based stigma, or negative PrEP perceptions [29, 76, 77]. To increase the likelihood of disclosure, AGYW should be attended to by a similar-aged, same-gender provider in a private location; in addition, providers should be given sensitivity training on AGYW’s sexuality, behavior, and risk. There must also be activities and dialogue to build mutual respect and trust between AGYW and providers. These implementation bottlenecks and barriers necessitate additional insight into other client- and provider-side factors that affect AGYW’s ability to make informed PrEP decisions and providers’ capability to offer quality services [78].

PEP use was a correlate of PrEP consultations in AG and YW. However, due to PEP and PrEP’s inherent clinical ties, it is difficult to isolate the effect of PEP use. Per MOH guidelines, PEP users who are seronegative at the end of the medication regimen are to immediately transition to PrEP [45]; it is also possible that AGYW sought out PrEP services but were, instead, prescribed PEP due to a recent high-risk encounter. This temporal ambiguity also complicates the finding that multiple partnerships was correlated with PrEP consultations among YW (i.e., did YW with multiple partners seek out PrEP services, or does PEP mediate some of the pathways between multiple partnerships and PrEP consultations?) Alternatively, YW with multiple partners could have frequented clinics/visited providers more often for HIV testing or other services, increasing their chances of engaging with providers about PrEP. Although we cannot disentangle these relationships, this PEP-PrEP connection suggests that AGYW with an existing linkage to the healthcare system were more likely to report PrEP consultations. Due to structural and community barriers, accessing healthcare systems can be precarious for AGYW [29, 79, 80]. Rather than have it siloed in clinics, PrEP services might be best delivered using community-based approaches—such as mobile or pop-up clinics—that bring care to areas that AGYW frequent.

When we examined PrEP eligibility criteria cumulatively, we discovered that AG and YW with > 1 criteria were more likely to report PrEP consultations than those with 1 criterion. While it is encouraging that AGYW with multiple vulnerabilities reported more consultations, this finding also implies that unless multiple risk factors were present, PrEP consultations were relatively rare. Moreover, it is possible that these criteria were not contemporaneous and, instead, occurred at separate time points; thus, we cannot deduce how many criteria were met when AGYW interacted with providers. Regardless, since only one criterion is needed for PrEP eligibility, these findings highlight the need for improved risk identification tools, tactics, and approaches to help AGYW consider their HIV risk and PrEP need, as well as help providers effectively counsel AGYW on PrEP.

While our study provides interesting insights, it is not without limitations. First, we could have underestimated risk since we could not measure injection drug use. Secondly, our use of definitional substitutions for the MOH’s PrEP eligibility criteria could have led to misclassification, potentially biasing our results. Subsequent research should use the same verbiage and timeframe as the MOH’s guidelines. Thirdly, because we used self-reported data, we cannot over-look the possibility of social desirability and/or recall bias. Fourthly, due to cross-sectional data, we cannot assess when behaviors or experiences occurred, relative to each other and PrEP consultations. Future research should employ a longitudinal design to examine the temporality of risk factors. Fifthly, due to limitations with our questionnaire, we could not assess oral PrEP offers and initiations outside the context of DREAMS, and no questions asked about adherence or continuation. This limitation warrants additional research into the connection between PrEP eligibility, consultations, and outcomes along the PrEP cascade. Lastly, this analysis used data collected approximately one year after national PrEP scale up; though we assumed that PrEP services were readily available since Kisumu County was a pilot site, it is possible that access to PrEP services was limited. Future studies should consider illuminating service availability by using spatial analyses.

## Conclusions

While efficacious, oral PrEP’s potential is wasted if it is inaccessible to individuals at substantial risk of HIV acquisition. To understand AGYW’s access to PrEP, we examined their PrEP eligibility and reports of PrEP consultations. Almost all AGYW in our sample were eligible, yet most did not report engaging in a PrEP consultation, and consultations were lower among AG than YW. Correlates of PrEP consultations included PEP use for, both, AG and YW and multiple sexual partnerships for YW, only. While PrEP consultations increased as the number of criteria increased, consultations were low (i.e., < 50%) in most criteria totals. The limited number of reported PrEP consultations implies that AGYW’s access to PrEP was low, necessitating implementation improvements and tools or strategies to improve risk identification. Further, efforts must be taken to ensure that in high HIV-transmission settings, PrEP consultations are offered to all AGYW, increasing the chances that AGYW—no matter their number of vulnerabilities—have the opportunity to decrease their HIV acquisition risk.

## Supplementary Material

1783017_Sup_material

## Figures and Tables

**Table 1:** Definition of study measures

Ministry of health criterion	Definition using DREAMS data
*Ministry of health PrEP eligibility criteria*	
In a serodiscordant relationship and trying to conceive	Said they were trying to conceive and perceived their partner to be at risk for HIV or were unable to assess their partner’s HIV risk
Recurrent sex under influence of alcohol/recreational drugs	Respondent or their primary or secondary partner drank alcohol prior to sex in the last 12 months
Engaged in transactional sex	In the last 12 months, engaged in sex with a stranger or casual partner for financial or material support (e.g., money for children or family, somewhere to stay, transportation, cell phone, etc.)
Recurrent PEP use	Ever visited or had been visited by a health service or doctor of any kind to receive PEP
Sexual partner (s) are of unknown HIV status and are at high-risk for HIV (e.g., from high HIV burden setting)	Never learned the HIV status of their primary or secondary partner
Sex with more than 1 person	Had > 1 sex partners or a secondary partner in the last 12 months
Recent STI experience	Reported experiencing any STI symptoms (genital ulcers, vaginal discharge, painful urination, or genital warts) in the last 6 months
Ongoing IPV/GBV	Reported experiencing any of the following in the last 12 months: *Physical IPV:* slapped or had something thrown at them; pushed or shoved; hit with a fist or another dangerous object; kicked, dragged, beaten, choked, or burnt; or threatened with a gun, knife, or other weapon by a current or previous boyfriend or partner *Sexual IPV:* had sex or performed other sexual acts against their will because a current or previous boyfriend or partner used physical coercion, threats and intimation, or force *Non-partner sexual violence:* a person other than their boyfriend or partner perpetrated unwanted sex by using persuasion or force—whether successful or unsuccessful; forced sex while they were under the influence of drugs or alcohol and too impaired to consent or refuse; or forced sex by two or more men—when the respondent was either sober or under the influence of drugs or alcohol
Inconsistent or no condom use	Did not “always” use condoms when having sex with primary or secondary partners in the last 30 days or past 12 months or reported not using a condom at last sex with primary or secondary partners
Injection drug use with shared needles and/or syringes	Unable to measure because it was not asked in our survey
Characteristic	Definition
*Sample characteristics*	
Age	Categorized as adolescent girls (aged 15–19 years) and young women (aged 20–24 years) using baseline (i.e., round 1) age
Socioeconomic position	Using a count variable comprised of water source (1 = piped into home or used public tap/0 = retrieved from dug well or river), toilet sharing (1 = no/0 = yes), and flooring type (1 = finished/0 = natural or rudimentary), we categorized socioeconomic position as low (count = 0), medium (count = 1 or 2), and high (count = 3). Because so few participants were in the high category (< 6% of sample), we combined the medium and high categories and analyzed socioeconomic position as low vs. medium/high
Orphanhood status	Never orphaned vs. at least one deceased parent
Marital status	Never vs. ever married
Pregnancy status	Never vs. ever pregnant
School enrollment	Not enrolled vs. currently enrolled
Perceived HIV risk	Using responses related to their likelihood of HIV exposure, we coded perceived risk as low (unlikely, not at all) vs. high (highly likely, somewhat likely, do not know)
Travel outside the community	Once a year or less vs. once a month or more
Location	Peri-urban vs. Urban study site
Travel time to clinic/hospital	From home, takes < 15 min, 15–29 min, 30–44 min, 45–59 min, or ≥ 60 min to get to the public clinic or hospital they typically go to for care. This variable acted as a proxy for service access and was only examined when analyzing the outcome
Outcome	
PrEP consultations	Ever visited a health service or been visited by a doctor to receive PrEP services or had a doctor or nurse talk to them about PrEP the last time they visited a healthcare provider or facility

*PrEP* pre-exposure prophylaxis, *DREAMS* determined, resilient, empowered, AIDS-free, mentored, safe, *PEP* post-exposure prophylaxis, *STI* sexually transmitted infection, *IPV* intimate partner violence, *GBV* gender-based violence

**Table 2:** Proportion of HIV negative, sexually active AGYW who met Kenya Ministry of Health’s PrEP Eligibility Criteria in Kisumu County, Kenya

	Adolescent girls (n = 154) n (%)	Young women (n = 289) n (%)	Chi-square test statistic	p value
Trying to conceive	0 (0.00)	6 (2.08)	^a^	0.097^b^
Alcohol/drug use during sex	4 (2.60)	29 (10.03)	8.06	0.005
Transactional sex	14 (9.09)	29 (10.03)	0.10	0.749
Recurrent PEP use	16 (10.39)	36 (12.46)	0.41	0.520
Unaware of partner’s HIV status	19 (12.34)	31 (10.73)	0.26	0.610
Multiple partners	21 (13.64)	43 (14.88)	0.13	0.723
Recent STI	33 (21.43)	64 (22.15)	0.03	0.862
Ongoing IPV/GBV	60 (38.96)	106 (36.68)	0.22	0.636
Inconsistent/no condom use	114 (74.03)	258 (89.27)	17.36	p < 0.001
At least one criterion	139 (90.26)	272 (94.12)	2.23	0.135

*AGYW* adolescent girls and young women, *PrEP* pre-exposure prophylaxis, *PEP* post-exposure prophylaxis, *STI* sexually transmitted infection, *IPV* intimate partner violence, *GBV* gender-based violence

a Data omitted because the Fisher’s Exact Test does not have a corresponding test statistic

b p value generated using Fisher’s Exact Test

**Table 3:** Intra- and intergroup characteristic differences of PrEP-ineligible and -eligible AGYW

	Adolescent girls					Young women				
	All (n= 154) n (%)	PrEP-ineligible (n = 15) n (%)	PrEP-eligible (n= 139) n (%)	Chi-square test statistic	p value	All (n = 289) n *(%)*	PrEP-ineligible (n = 17) n (%)	PrEP-eligible (n = 272) n (%)	Chi-square test statistic	p value
Socioeconomic position										
Low	23 (14.94)	0 (0.00)	23 (16.55)			44 (15.22)	2 (11.76)	42 (15.44)		
Middle/High	131 (85.06)	15 (100.00)	116 (83.45)	^a^	0.129^b^	245 (84.78)	15 (88.24)	230 (84.56)	^a^	1.000^b^
Orphanhood										
Never	90 (58.44)	9 (60.00)	81 (58.27)			120 (41.52)	6 (35.29)	114 (41.91)		
Ever	64 (41.56)	6 (40.00)	58 (41.73)	0.02	0.897	169 (58.48)	11 (64.71)	158 (58.09)	0.29	0.591
Married										
Never	131 (85.06)	15 (100.00)	116 (83.45)			140 (48.44)	16 (94.12)	124 (45.59)		
Ever	23 (14.94)	0 (0.00)	23 (16.55)	^a^	0.129^b^	149 (51.56)	1 (5.88)	148 (54.41)	15.09	p<0.001
Pregnant										
Never	98 (63.64)	13 (86.67)	85 (61.15)			64 (22.15)	12 (70.59)	52 (19.12)		
Ever	56 (36.36)	2 (13.33)	54 (38.85)	3.81	0.051	225 (77.85)	5 (29.41)	220 (80.88)	^a^	p<0.001^b^
School enrollment										
Not enrolled	70 (45.45)	4 (26.67)	66 (47.48)			220 (76.12)	7 (41.18)	213 (78.31)		
Currently enrolled	84 (54.55)	11 (73.33)	73 (52.52)	2.37	0.124	69 (23.88)	10 (58.82)	59 (21.69)	^a^	0.002^b^
Perceived HIV Risk										
Low	120 (77.92)	15 (100.00)	105 (75.54)			212 (73.36)	13 (76.47)	199 (73.16)		
High	34 (22.08)	0 (0.00)	34 (24.46)	^a^	0.043^b^	77 (26.64)	4 (23.53)	73 (26.84)	^a^	1.000^b^
Travel outside the community										
Once a year or less	122 (79.22)	13 (86.67)	109 (78.42)			208 (71.97)	11 (64.71)	197 (72.43)		
Once a month or more	32 (20.78)	2 (13.33)	30 (21.58)	^a^	0.738^b^	81 (28.03)	6 (35.29)	75 (27.57)	^a^	0.578^b^
Location										
Peri-Urban	77 (50.00)	7 (46.67)	70 (50.36)			138 (47.75)	10 (58.82)	128 (47.06)		
Urban	77 (50.00)	8 (53.33)	69 (49.64)	0.07	0.786	151 (52.25)	7 (41.18)	144 (52.94)	0.89	0.346

*PrEP* pre-exposure prophylaxis, *AGYW* adolescent girls and young women

a Data omitted because the Fisher’s Exact Test does not have a corresponding test statistic

b *p* value generated using Fisher’s Exact Test

**Table 4:** Unadjusted and adjusted analyses to identify knowledge, behavioral, and experiential correlates of PrEP consultations among PrEP-eligible AGYW

	Adolescent girls					Young women				
	No (n= 105) n (%)	Yes (n = 34) n (%)	*z* test statistic^a^	p value^a^	aPR (95% Cl)	No (n= 160) n (%)	Yes (n= 112) n (%)	Chi-square test statistic	p value	aPR (95% CI)
*Characteristics*										
Socioeconomic position										
Low	19 (82.61)	4 (17.39)				21 (50.00)	21 (50.00)			
Middle/High	86 (74.14)	30 (25.86)	− 0.84	0.399		139 (60.43)	91 (39.57)	1.60	0.206	
Orphanhood										
Never	65 (80.25)	16 (19.75)				63 (55.26)	51 (44.74)			
Ever	40 (68.97)	18 (31.03)	1.52	0.128		97 (61.39)	61 (38.61)	1.03	0.311	
Married										
Never	90 (77.59)	26 (22.41)				77 (62.10)	47 (37.90)			
Ever	15 (65.22)	8 (34.78)	1.35	0.177		83 (56.08)	65 (43.92)	1.01	0.315	
Pregnant										
Never	66 (77.65)	19 (22.35)				36 (69.23)	16 (30.77)			
Ever	39 (72.22)	15 (27.78)	0.76	0.449		124 (56.36)	96 (43.64)	2.88	0.090	
School enrollment										
Not enrolled	46 (69.70)	20 (30.30)				130 (61.03)	83 (38.97)			
Currently enrolled	59 (80.82)	14 (19.18)	− 1.53	0.126		30 (50.85)	29 (49.15)	1.98	0.160	
Perceived HIV risk										
Low	78 (74.29)	27 (25.71)				122 (61.31)	77 (38.69)			
High	27 (79.41)	7 (20.59)	− 0.59	0.555		38 (52.05)	35 (47.95)	1.89	0.170	
Travel outside the community										
Once a year or less	87 (79.82)	22 (20.18)				123 (62.44)	74 (37.56)			
Once a month or more	18 (60.00)	12 (40.00)	2.36	0.018		37 (49.33)	38 (50.67)	3.85	0.050	
Location										
Peri-urban	53 (75.71)	17 (24.29)				81 (63.28)	47 (36.72)			
Urban	52 (75.36)	17 (24.64)	0.05	0.960		79 (54.86)	65 (45.14)	1.98	0.159	
Travel time to clinic/hospital										
< 15 min	19 (76.00)	6 (24.00)				31 (64.58)	17 (35.42)			
15–29 min	33 (75.00)	11 (25.00)	0.09	0.928		44 (53.66)	38 (46.34)			
30–44 min	26 (74.29)	9 (25.71)	0.15	0.881		51 (64.56)	28 (35.44)			
45–59 min	11 (78.57)	3 (21.43)	− 0.18	0.853		14 (63.64)	8 (36.36)			
≥60 min	16 (76.19)	5 (23.81)	− 0.02	0.982		20 (48.78)	21 (51.22)	4.55	0.337	
*Ministry of health PrEP eligibility criteria*										
Trying to conceive in a serodiscordant relationship										
No	105 (75.54)	34 (24.46)				157 (59.02)	109 (40.98)			
Yes	0 (0.00)	0 (0.00)	^b^	^b^		3 (50.00)	3 (50.00)	^c^	0.693^d^	
Recurrent sex with alcohol/drugs										
No	103 (76.30)	32 (23.70)				143 (58.85)	100 (41.15)			
Yes	2 (50.00)	2 (50.00)	1.42	0.156		17 (58.62)	12 (41.38)	<0.01	0.981	
Engaged in transactional sex										
No	93 (74.40)	32 (25.60)				148 (60.91)	95 (39.09)			-
Yes	12 (85.71)	2 (14.29)	− 0.86	0.390		12 (41.38)	17 (58.62)	4.08	0.043	-
Recurrent PEP use										
No	104 (84.55)	19 (15.45)			Ref	159 (67.37)	77 (32.63)			Ref
Yes	1 (6.25)	15 (93.75)	8.15	<0.001	5.63 (3.53–8.97)	1 (2.78)	35 (97.22)	53.81	<0.001	2.81 (2.30–3.43)
Do not know partner(s) HIV status										
No	93 (77.50)	27 (22.50)				146 (60.58)	95 (39.42)			
Yes	12 (63.16)	7 (36.84)	1.42	0.155		14 (45.16)	17 (54.84)	2.70	0.101	
Sex with more than 1 person										
No	91 (77.12)	27 (22.88)				144 (62.88)	85 (37.12)			Ref
Yes	14 (66.67)	7 (33.33)	1.07	0.286		16 (37.21)	27 (62.79)	9.85	0.002	1.39 (1.06–1.82)
Recent STI										
No	81 (76.42)	25 (23.58)				127 (61.06)	81 (38.94)			
Yes	24 (72.73)	9 (27.27)	0.43	0.667		33 (51.56)	31 (48.44)	1.82	0.177	
Ongoing IPV/GBV										
No	62 (78.48)	17 (21.52)				100 (60.24)	66 (39.76)			
Yes	43 (71.67)	17 (28.33)	0.92	0.356		60 (56.60)	46 (43.40)	0.35	0.552	
Inconsistent/no condom use										
No	18 (72.00)	7 (28.00)				8 (57.14)	6 (42.86)			
Yes	87 (76.32)	27 (23.68)	− 0.46	0.643		152 (58.91)	106 (41.09)	0.02	0.896	

Row percentages are presented

A dash (−) represents eligibility criteria that were included in the initial adjusted model but were removed during the backward selection process

For AG, the adjusted model (aPR column) included travel outside the community and program targeting

*PrEP* pre-exposure prophylaxis, *AGYW* adolescent girls and young women, *aPR* adjusted prevalence ratio, *CI* confidence intervals, *PEP* post-exposure prophylaxis, ref reference category, *STI* sexually transmitted infection, *IPV* intimate partner violence, *GBV* gender-based violence

a Test statistic and p values were generated using a model that controlled for differential program targeting

b Test statistic and p value cannot be calculated due to zero respondents in one category

c Data omitted because the Fisher’s Exact Test does not have a corresponding test statistic

d p value based on Fisher’s Exact Test

## Data Availability

All the data are available on Dataverse.
