# Supplementary material for: Oral PrEP Consultations Among Adolescent Girls and Young Women in Kisumu County, Kenya: Insights from the DREAMS Program
Source: AIDS Behav. Author manuscript; Available in PMC 2022 Aug 1. (PMC9252953; doi:10.1007/s10461-022-03590-z)
Supplement: 1783017_Sup_material [file NIHMS1783017-supplement-1783017_Sup_material.docx]

**Supplemental Table I. Spearman Correlation Coefficients and Variance Inflation Factors (VIF) of Independent Variables used in Multivariable models**

| **Table IA.** | **Assessing multicollinearity of multivariable Model I** | | | |  | | |
| --- | --- | --- | --- | --- | --- | --- | --- |
|  | Ever married | Ever pregnant | Enrolled in school | VIF |  |  |  |
|  |  |  |  |  |  |  |  |
| Ever married | 1.000 |  |  | 1.38 |  |  |  |
|  |  |  |  |  |  |  |  |
| Ever pregnant | 0.500 | 1.000 |  | 1.35 |  |  |  |
|  | p<0.001 |  |  |  |  |  |  |
| Enrolled in school | -0.269 | -0.229 | 1.000 | 1.09 |  |  |  |
|  | p<0.001 | p<0.001 |  |  |  |  |  |
|  |  |  |  |  |  |  |  |
| **Table IB.** | **Assessing multicollinearity of multivariable Model II** | | | | |  |  |
|  | Ever orphaned | Ever married | Ever pregnant | Enrolled in school | VIF |  |  |
|  |  |  |  |  |  |  |  |
| Ever orphaned | 1.000 |  |  |  | 1.07 |  |  |
|  |  |  |  |  |  |  |  |
| Ever married | 0.130 | 1.000 |  |  | 1.49 |  |  |
|  | p=0.008 |  |  |  |  |  |  |
| Ever pregnant | 0.124 | 0.545 | 1.000 |  | 1.50 |  |  |
|  | p=0.012 | p<0.001 |  |  |  |  |  |
| Enrolled in school | -0.254 | -0.369 | -0.376 | 1.000 | 1.28 |  |  |
|  | p<0.001 | p<0.001 | p<0.001 |  |  |  |  |
|  |  |  |  |  |  |  |  |
| **Table IC.** | **Assessing multicollinearity of multivariable Model III** | | | | | | |
|  | Age group | Ever orphaned | Ever married | Ever pregnant | Enrolled in school | Program targeting | VIF |
|  |  |  |  |  |  |  |  |
| Age group | 1.000 |  |  |  |  |  | 1.52 |
|  |  |  |  |  |  |  |  |
| Ever orphaned | 0.155 | 1.000 |  |  |  |  | 1.08 |
|  | p=0.002 |  |  |  |  |  |  |
| Ever married | 0.363 | 0.130 | 1.000 |  |  |  | 1.52 |
|  | <0.001 | p=0.008 |  |  |  |  |  |
| Ever pregnant | 0.422 | 0.124 | 0.545 | 1.000 |  |  | 1.61 |
|  | p<0.001 | p=0.012 | p<0.001 |  |  |  |  |
| Enrolled in school | -0.312 | -0.254 | -0.369 | -0.376 | 1.000 |  | 1.32 |
|  | p<0.001 | p<0.001 | p<0.001 | p<0.001 |  |  |  |
| Program targeting | 0.491 | 0.135 | 0.249 | 0.314 | -0.293 | 1.000 | 1.37 |
|  | p<0.001 | p=0.006 | p<0.001 | p<0.001 | p<0.001 |  |  |
|  |  |  |  |  |  |  |  |
| **Table ID.** | **Assessing multicollinearity of multivariable Model IV** | | | |  |  |  |
|  | Ever used PEP | Travel outside community | Program targeting | VIF |  |  |  |
|  |  |  |  |  |  |  |  |
| Ever used PEP | 1.000 |  |  | 1.04 |  |  |  |
|  |  |  |  |  |  |  |  |
| Mobility | 0.194 | 1.000 |  | 1.07 |  |  |  |
|  | p=0.022 |  |  |  |  |  |  |
| Program targeting | 0.009 | 0.176 | 1.000 | 1.03 |  |  |  |
|  | p=0.919 | p=0.038 |  |  |  |  |  |
|  |  |  |  |  |  |  |  |
| **Table IE.** | **Assessing multicollinearity of multivariable Model V** | | | |  |  |  |
|  | Transactional sex | Ever used PEP | Multiple partners | VIF |  |  |  |
|  |  |  |  |  |  |  |  |
| Transactional sex | 1.000 |  |  | 1.67 |  |  |  |
|  |  |  |  |  |  |  |  |
| Ever used PEP | 0.076 | 1.000 |  | 1.03 |  |  |  |
|  | p=0.212 |  |  |  |  |  |  |
| Multiple partners | 0.634 | 0.158 | 1.000 | 1.71 |  |  |  |
|  | p<0.001 | p=0.009 |  |  |  |  |  |
|  |  |  |  |  |  |  |  |
| **Table IF.** | **Assessing multicollinearity of multivariable Model VI** | | | |  |  |  |
|  | Ever used PEP | Multiple partners | VIF |  |  |  |  |
| Ever used PEP | 1.000 |  | 1.03 |  |  |  |  |
|  |  |  |  |  |  |  |  |
| Multiple partners | 0.158 | 1.000 | 1.03 |  |  |  |  |
|  | p=0.009 |  |  |  |  |  |  |

PEP = Post-exposure prophylaxis

P-values of Spearman’s Correlation are located under each coefficient
